# Why are so many MLL lysine methyltransferases required for normal mammalian development?

**DOI:** 10.1007/s00018-019-03143-z

**Published:** 2019-05-16

**Authors:** Nicholas T. Crump, Thomas A. Milne

**Affiliations:** MRC Molecular Haematology Unit, MRC Weatherall Institute of Molecular Medicine, NIHR Oxford Biomedical Research Centre Haematology Theme, Radcliffe Department of Medicine, University of Oxford, Oxford, UK

**Keywords:** MLL, Methylation, Histone, H3K4, Transcription, Epigenetics, Development

## Abstract

The mixed lineage leukemia (MLL) family of proteins became known initially for the leukemia link of its founding member. Over the decades, the MLL family has been recognized as an important class of histone H3 lysine 4 (H3K4) methyltransferases that control key aspects of normal cell physiology and development. Here, we provide a brief history of the discovery and study of this family of proteins. We address two main questions: why are there so many H3K4 methyltransferases in mammals; and is H3K4 methylation their key function?

## Background

The *mixed lineage leukemia* (*MLL*) gene was originally identified in humans due to its association with a common breakpoint found in a subset of incurable acute leukemias [1–4]. Most *MLL* gene mutations in leukemia are chromosome translocations that truncate the *MLL* gene and fuse it in frame to an ever-increasing number of different partner genes [5]. The role of *MLL* mutations in leukemia has been extensively explored in multiple reviews over the years [6–9]. Here, we focus on what is known about the function of the wild-type MLL protein and its related family members, a perhaps less studied but in some ways even more complicated topic.

## History

When the human *MLL* cDNA was first sequenced [2–4], it was found to have a striking homology to the *Drosophila* gene *trithorax* [10]. Trithorax (or trx) is a founding member of the trithorax group (trxG) of proteins [11, 12] which were originally identified as regulators of *Homeotic* (or *Homeobox*, *Hox*) genes in *Drosophila*, a set of genes that are essential for body patterning in multi-cellular organisms [11–15]. TrxG proteins maintain the expression of *Hox* genes and this is antagonized by the repressive activity of the Polycomb group (PcG) of proteins [13, 15, 16]. Importantly, neither group of regulators is required for initiating *Hox* gene expression, but instead they are required to maintain expression patterns once they have been established by early acting transcription factors [15, 17]. Interestingly, both PcG and trxG genes were discovered in parallel with the *Hox* genes, due to the overall importance of this entire system for controlling development of the anterior–posterior axis [11–15]. Seminal work in mice showed that mammalian *Mll* behaves like a member of the trxG, in that *Mll* knockout (KO) mice display embryonic lethality and body plan defects (Embryonic day 9; E9) caused by altered *Hox* gene expression patterns [18, 19]. A key aspect of this work is that, similar to observations with *trx* mutants, *Hox* gene expression patterns initiate normally in *Mll* mutants but only break down at later stages of development [18, 19].

The mutually antagonistic nature of PcG and trxG function was highlighted by the observation that PcG/trxG double mutants in both *Drosophila* [20–22] and mice [23] produced embryos that were phenotypically closer to wild type than either individual mutation. Further support of this model came from work that revealed another class of genes, the so-called “enhancers of trithorax and Polycomb” (ETPs, [24]). For example, the *Drosophila* gene *Additional sex combs* (*Asx*) is a key member of this group [25] whose ETP function may be conserved in mammals [26]. Mutations in ETP genes enhance the activity of both PcG and trxG mutations, but alone they display phenotypes closer to wild type [25, 27].

Despite the early observations that PcG and trxG proteins balanced each other out and were required for maintenance but not initiation of gene expression, the genetics alone did not reveal any clues to the function of these proteins on a molecular level. A key aspect of this came from work on the SET domain.

## The SET domain and methyltransferase activity

SET is an acronym [28] taken from the founder members of this family: Suppressor of variegation 3-9 (Su(var)3-9) [28]; Enhancer of Zeste (E(z)) [29]; and Trithorax [30]. All three are chromatin proteins, with Su(var)3-9 promoting repressive heterochromatin and E(z) and trx being members of the PcG and trxG, respectively. What was interesting to the field at the time was that these proteins, with apparently quite different functions, all contained a homologous protein domain indicating a possible similar activity. Using sequence homology, the SET domain was found in over 140 genes in multiple species including plants, bacteria and some viruses [31, 32]. The recognition that this domain was present in some plant *N*-methyltransferases led to the discovery that the SET domain in mammals was a lysine methyltransferase (KMT), capable of methylating lysine residues on histones [31].

Methylation had long been known to occur on histone proteins [33], including on specific lysine residues in a mono-, di- or tri-methyl form (reviewed in: [34–36]). A key discovery in understanding the distinct functions of different SET domain-containing proteins was that these domains had specificity for different lysine residues. For instance, EZH2, the mammalian homolog of the PcG protein E(z), specifically methylates lysine 27 on histone H3 (H3K27) [37] while SUV39H1, homolog of heterochromatic protein Su(var)3-9, methylates lysine 9 on histone H3 (H3K9) [31]. Interestingly, a viral SET domain-containing protein was identified with intrinsic H3K27 methyltransferase capabilities, raising the possibility that viral proteins may be able to alter the epigenome of the host [38].

**Fig. 1:**
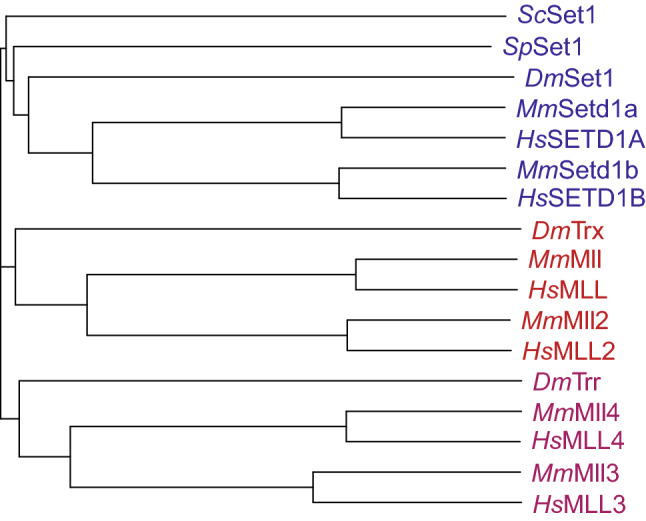
Sequence conservation of MLL family proteins. Evolutionary distances between MLL family protein sequences in human (Hs), mouse (Mm), *Drosophila* (Dm), *Saccharomyces cerevisiae* (Sc) and *Schizosaccharomyces pombe* (Sp), calculated using ClustalW. Horizontal lengths are proportional to sequence similarity distance

Surprisingly, in contrast to SUV39H1 and EZH2, the MLL SET domain was initially thought to be functionally inactive [31]. However, subsequent work showed that it had methyltransferase activity specific for lysine 4 on histone H3 (H3K4) [39, 40], a modification known to be associated with active genes. H3K4 methyltransferase activity was found to be targeted directly to *Hox* genes by the full-length MLL protein and this resulted in the activation of their expression [39]. Previous work had identified Set1 in *Saccharomyces cerevisiae* as the major H3K4 methyltransferase in yeast [41–44], leading some to suggest that Set1 could be the yeast homolog of MLL/trx [45, 46]. However, subsequent work identified mammalian equivalents of Set1 [47–49], suggesting that although MLL and *S. cerevisiae* Set1 were related in their SET domains, the MLL protein had likely evolved to also take on other functions. In addition, the discovery of other MLL/trx-like SET domain-containing genes in *Drosophila* such as *trithorax*-*related* (*trr*) [50, 51] and *Drosophila*-*Set1* (*dSet1*) [52, 53] indicated that MLL-like genes may represent a specific group in higher organisms that had diverged from the original Set1 protein in yeast (Fig. 1). This point is discussed in more detail below.

In the original studies that demonstrated MLL H3K4 methyltransferase activity, neither the purified SET domain [39] nor a purified MLL complex [40] was able to deposit trimethylation (H3K4me3). However, later work showed that a different MLL complex preparation actually had a preference for trimethylating H3K4, and this was associated with elevated H3K4me3 at *Hox* genes and stimulation of transcription from an in vitro chromatin template [54]. The apparently contradictory results were clarified by the observation that the MLL SET domain requires three cooperating proteins for full methyltransferase activity: WDR5, RbBP5 and ASH2L [55]. These components were not identified in the original MLL complex purification [40], but it is now known that WDR5, RbBP5 and ASH2L are common components of all H3K4me3 SET domain complexes, something that is also shared with Set1 in yeast [56].

Overall, the major importance of the SET domain work was that it provided a possible functional explanation for the antagonism of PcG vs trxG proteins. The ability to methylate specific lysine residues could help promote either gene activation (H3K4me3) or repression (H3K27me3), thus it was initially thought that the main function of these proteins was mostly explained by SET domain activity. Differential SET domain function also provides a possible functional explanation for the observation that the ETP Asx/ASXL1 is required for both trxG and PcG function [25, 26]. Asx has been found to interact with both the E(z) and Trx protein SET domains, controlling both activation and repression by modulating H3K4me3 and H3K27me3 levels at target loci [57]. However, the Asx family co-purifies in a complex containing the deubiquitinase BAP1 rather than EZH2 or MLL in both *Drosophila* and mammals [58], suggesting that further characterization of these interactions is required.

Despite these exciting observations, the fact that deletion of the MLL SET domain resulted in viable and fertile mice, albeit with developmental skeletal defects and disturbed *Hox* gene expression [59, 60], argued that the SET domain alone could not account for the main function of the MLL protein.

## The MLL family

**Fig. 2:**
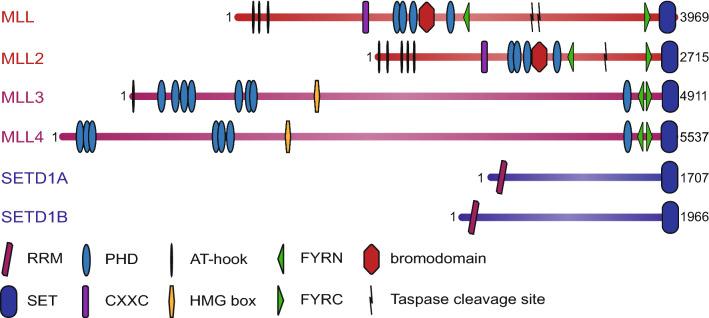
Domain structure of mammalian MLL family proteins. The six human MLL family proteins are shown, with the approximate positions and sizes of identified domains. Numbers indicate the length of each protein. All contain a C-terminal SET domain which catalyses histone H3K4 methylation, as well as a variable number of DNA-binding and protein–protein interaction domains. RRM: RNA-recognition motif; PHD: plant homeodomain; FYRN/FYRC: FY-rich domain, N-/C-terminal; SET: Su(var)3-9/E(z)/Trithorax domain; HMG box: high mobility group box; Taspase cleavage site: recognition sequence cleaved by the threonine protease Taspase 1 Adapted from [56]

There are six members of the “MLL family” in mammals [56, 61]. The family is made up of three pairs of highly structurally-related proteins, with each pair related to a single *Drosophila* protein (Fig. 1) [56, 62]. There has been some confusion about the naming of the individual members, especially MLL2/4 [7], but we adopt the convention here of referring to the human gene at 19q13 as MLL2/KMT2B, which fits best with the structural relatedness of the proteins (see Figs. 1 and 2) as further described below [61]. MLL (MLL1 or KMT2A [1], human chromosome 11q23) pairs with MLL2 (KMT2B [63, 64], human chromosome 19q13) and these are both most closely related to trx itself [2–4, 10, 50, 62]. MLL3 (KMT2C, human chromosome 7q36 [65, 66]) pairs with MLL4 (KMT2D, human chromosome 12q13 [67, 68]) and these are highly related to the trithorax-related (trr) protein [50, 62]. Finally, SETD1A (KMT2F human chromosome 16p11 [47]) pairs with SETD1B (KMT2G human chromosome 12q24 [49]) and these are both closest to the *Drosophila* Set1 protein (dSet1) which in turn is the closest homolog to *S. cerevisiae* Set1 (Fig. 1) [52, 53, 62]. Although MLL5 (KMT2E [69]) was originally thought to be a member of the MLL family, the lack of intrinsic KMT activity and the observation that the MLL5 SET domain is divergent from the rest of the family has led to it being reclassified as representing a different subgroup of SET domain proteins [70].

The main commonality between the MLL family members is that they share a highly related SET domain that is capable of methylating H3K4 [39, 47, 71–73]. MLL family members also all interact with the complex components WDR5, RbBP5, ASH2L and DPY30, which represent a core set of proteins required for full KMT activity of the SET domain [56, 62]. However, beyond the SET domain, different MLL family members display a different protein domain architecture ([56, 62] and see Fig. 2). Furthermore, despite residing in overlapping protein complexes, in some cases individual members can interact with unique sets of proteins, although interacting factors and unique domains are often shared between each of the partner pairs [7, 56]. Thus, although the MLL proteins are a “family” from the perspective that they represent an evolutionary and functional expansion of the SET1 system, it is worth considering that there are multiple other mammalian H3K4 KMTs (with less closely related KMT domains) such as SET7/9 [74] and the SMYD family of proteins [75, 76].

Since the cell has multiple ways of directing H3K4 KMT activity beyond the MLL family, it is probably more accurate to think of the SET domain as providing only a subset of each member’s function. This perspective fits with the observation that loss of the MLL SET domain does not phenocopy deletion of the entire gene [59, 77]. Following this logic, we would argue that these proteins, which happen to share H3K4 KMT activity and a similar evolutionary origin, are functionally distinct with unique biological roles in the cell, much in the same way an artist and a decorator may both use a paintbrush to different ends in their jobs. For this reason, the rest of this review will focus on the potentially unique roles of these proteins, how the individual proteins may be specifically directed towards key targets in the cell, and how their KMT activity may contribute to their overall function.

## Function of H3K4 methylation

Before discussing MLL family member-specific activities, it is worth briefly touching on what the general role of H3K4 methylation is thought to be. Some of the earliest work showed that H3K4 methylation was highly associated with transcriptionally active *Tetrahymena* macronuclei [78]. This fit with later observations that indicated increased H3K4me2/3 was associated with elevated *Hox* gene expression as well as increased in vitro transcription on nucleosomal templates [39, 54, 79–81]. Yeast Set1 is recruited by the RNA polymerase II (RNA pol II) elongation machinery to target H3K4 methylation to actively transcribed genes [82]. Early experiments indicated that MLL could also associate with elongating RNA pol II [83], but more recent interactome studies with differentially phosphorylated versions of the RNA pol II C-terminal domain (CTD) failed to co-purify MLL family complex components [84]. Notably, the lack of correlation between MLL and RNA pol II binding within the gene body [85] also indicates that if this interaction ever occurs in vivo it is not sufficient for localisation.

More detailed analyses of the genome-wide distribution of H3K4 methylation supported these initial observations of a role in gene regulation but also suggested a slightly more nuanced pattern of activity based on the precise methylation status. H3K4me1 was found to be primarily associated with enhancers, and H3K4me2 was more generally distributed throughout active genes and enrichment for H3K4me3 marked active promoters [86–91]. The discovery of multiple protein domains possessing the ability to bind directly to methylated lysine residues provided a major functional insight into how H3K4me3 could impact promoter function. Examples of tandem chromodomains [92, 93], Plant HomeoDomain (PHD) zinc finger motifs [94] and Tudor domains [95–97], have been identified with specificity for H3K4me3, linking a number of protein activities with this modification. For example, the PHD motif is found in multiple proteins [56] thought to promote gene activation including nucleosome remodelers [56, 94, 98] and TAF3, a component of the RNA pol II pre-initiation complex [56, 79, 99]. Elegant in vitro studies indicated that TAF3 binding to H3K4me3 substrates could stimulate transcription by increasing the stability of the pre-initiation complex, allowing for rapid re-initiation through multiple rounds of transcription [79]. In contrast to the incredible range of proteins that bind to H3K4me3, proteins that specifically recognize H3K4me1 have been harder to find [56], although recent work suggests that the cohesin complex may be stabilized by binding to H3K4me1 [100]. However, the specifics of this interaction have yet to be established.

Despite the strong data linking H3K4 methylation to both enhancer and promoter function, *SET1* KO or H3K4 point mutations that completely abolish H3K4me2/3 in yeast have a relatively mild phenotype [41]. In general, global loss of H3K4me3 induces very few transcriptional changes in most systems [101–103]. In addition, despite the fact that the breadth and height of H3K4me3 peaks correlate with higher levels of gene expression [104, 105], more recent work in mouse embryonic stem (ES) cells suggests that even where there are gene expression changes, there is very little correlation between the reduction in H3K4me3 levels and the extent of gene expression changes, at least globally [106]. Instead, the requirement for H3K4me3 appears to be gene specific, with the dependence on H3K4me3 appearing to be stronger at genes where there are potentially fewer “activating signals” maintaining expression. In addition, H3K4me1 appears to be dispensable for enhancer function in ES cells [107] and throughout *Drosophila* development [108]. These and other observations have led to the question of whether H3K4 methylation is actually required for promoting transcription or enhancer function, whether it has a much more subtle role in modulating gene expression, or even if it is simply a non-functional marker of past transcription [103]. Given the implied significance of the MLL family SET domains, this has important potential implications for their function.

## Distinct biological phenotypes of MLL family members

### MLL/MLL2

As described above, knockouts of the *Mll* gene in mice produced antero-posterior skeletal defects highly reminiscent of the *Hox* gene-mediated body pattern defects observed in *trx* mutant embryos [18, 19, 23]. Since embryonic death occurred relatively early, a full analysis of hematopoietic defects was not possible, but initial analysis of KO models indicated that MLL likely has a key role in normal hematopoietic development, primarily through the regulation of *Hox* genes [109–111]. Inducible *Mll* KO models were produced to more directly address MLL function in fetal and adult hematopoiesis, and although there were some specific differences between the KO models, in general MLL was found to be required for normal hematopoietic development [112–114]. These MLL phenotypes are contrasted by the observation that deletion of the MLL SET domain has very little impact on embryogenesis [59] or hematopoiesis [60]. Since loss of MLL has profound effects on gene expression, especially of the *Hox* genes [39, 60, 115], gene activation as mediated by MLL may require other domains of the protein (discussed below), likely by recruiting components of the MLL complex, such as MOF-mediated H4K16 acetylation (H4K16ac) [54, 60]. Interestingly, however, this work contrasts with what is observed in *Drosophila* where a point mutation in the SET domain of trx is sufficient to produce trx-dependent developmental defects such as homeotic phenotypes [116]. This could be due to the fact that there is some redundancy in the mammalian system between the different MLL family members, at least in terms of H3K4me2/3 deposition.

Despite the fact that MLL and MLL2 are quite similar proteins, KOs of *Mll2* display a much more severe phenotype that result in early embryonic lethality with widespread evidence of apoptosis [61]. No body patterning defects were observed, but this could be due to the severity of the early-stage phenotype [61]. Interestingly, loss of MLL2 did have an impact on *Hox* gene expression, but mostly members of the *HoxB* cluster, whereas MLL primarily affects *HoxA* and *C* cluster genes [39, 61, 83]. Another major difference is that an inducible *Mll2* KO has no effect on adult tissues or on normal blood development [117], except perhaps for a highly specific phenotype in macrophages [118]. Knockdown of MLL2 in mouse ES cells results in a reduction in H3K4me3 levels primarily at bivalent genes (whose promoters are marked with both H3K4me3 and H3K27me3), suggesting that MLL2 has a specific function at these genes [119, 120], although it is not clear whether this function is exclusive to MLL2. Notably, knockdown of MLL2 does not appear to disrupt the transcriptional changes at bivalent genes induced by retinoic acid (RA) [119, 120], even though the MLL2-associated factor AKAP95 is required for RA-mediated gene induction in ES cells [81].

### MLL3/MLL4

Mutation of trr, the *Drosophila* homolog of MLL3/4, displays a vastly different phenotype to trx. Unlike *trx*, *trr* mutants do not display *Hox* gene defects or interact with either PcG or trxG mutations [50]. Instead, trr acts in retinal development and promotes hormone receptor-mediated gene activation [51] as well as functioning in the suppression of tissue growth [121].

The extent to which MLL3 and MLL4 play different roles in the cell is unclear. MLL4 has been shown to be only partly independent of MLL3 in adipogenesis and myogenesis [122]. *Mll3* KO mice die at birth with no obvious morphological abnormalities, whereas an *Mll4* KO results in embryonic lethality around day E9.5 [122], comparable to the *Mll* KO mouse, although whether *Mll4* KOs affect *Hox* gene expression patterns has not been studied. Unlike MLL2, MLL4 can impact RA-regulated genes [123]. There is also an association of MLL3/4 mutations with some leukemias [124], and consistent with this both MLL3 and MLL4 are required for normal blood stem and progenitor cell function [125, 126].

### SETD1A/SETD1B

dSet1 is responsible for most of the H3K4me3 in *Drosophila* [52, 127] and as previously mentioned is the closest relative to yeast Set1 (Fig. 1). Despite their high level of similarity, both SETD1A and SETD1B are individually required for normal mouse embryogenesis and display quite different KO phenotypes [128]. *Setd1a* KO embryos display a severe phenotype and die before gastrulation, while *Setd1b* KO embryos survive until E11.5, albeit in a severely growth-retarded condition [128]. In line with the embryonic phenotypes, *Setd1a* KO ES cells stop proliferating and die, while ES cells tolerate the loss of *Setd1b* [128]. Interestingly, only *Setd1a* KO ES cells display a global loss of H3K4 methylation [128]. Inducible KO models indicate SETD1A has a role in B cell development [129] and in erythropoiesis [130], but otherwise a *Setd1a* deletion does not display hematopoietic defects.

Overall, there are profound phenotypic differences between MLL family members, even between the highly related gene pairs, which make it clear that each protein has a unique function in the organism. Since most MLL family members are ubiquitously expressed, the most common explanation for this is that their H3K4 methyltransferase activity is differentially targeted within the nucleus. There is some evidence for this (discussed below), but it cannot fully explain the vast differences in phenotype between the different family members. Instead, we argue the biological data support the idea that each protein has a distinct molecular function, and that KMT activity is not the sole or even the major molecular role of each protein. In particular, while KOs of the different MLL family members have profound impacts on normal development, this is not always associated with changes in H3K4 methylation. Additionally, even though it is the only member to have been directly tested, it is clear that loss of MLL SET domain activity does not accurately phenocopy loss of the entire protein during development.

## Distinct functional activities of MLL family members

### MLL3/4 and enhancer function

In common with MLL/MLL2, MLL3/4 were initially found to be important for regulating expression of a subset of target genes [123]. However, a broader role for these proteins has since been identified at gene enhancers, sequences distal to target promoters. Given the initial identification of Trx in *Drosophila*, it is perhaps fitting that its homolog Trr was first shown to deposit H3K4me1 at enhancers [131] before a similar role was identified for MLL3 and MLL4 [122, 132]. Consistent with this, in vitro MLL3/4 show a reduced ability to trimethylate H3K4, relative to mono- and dimethylation [80, 122, 133].

Deletion of MLL4 disrupts levels of many features associated with enhancers, including H3K4me1, H3K27ac, Mediator, RNA pol II and enhancer transcription (eRNAs) [107, 122, 134], and MLL3/4 KOs disrupt CBP/p300 binding at these loci [135, 136]. A key feature of active enhancers is their ability to interact with target gene promoters, and these contacts appear to be dependent on MLL3/4. Double KOs of *Mll3/4* in ES cells result in a reduction in short-range (< 100 kb) interactions, as measured by Hi-C, correlating with MLL3/4-dependent H3K4me1 loci [100]. Higher resolution analysis revealed specific loss of promoter–enhancer contacts for *Sox2* [100], *Nanog* and *Lefty* [135]. Loss of MLL3/4 also resulted in reduced Rad21 (a subunit of the cohesin complex) localisation to enhancers [100], suggesting a potential mechanism for disruption of promoter–enhancer interactions.

Taken together, these results argue for a key role for MLL3/4 in the establishment or maintenance of enhancers. Is MLL3/4 simply required for deposition of H3K4me1, or are the non-SET domains important for enhancer function? This issue has been addressed using an ES cell line in which SET domain point mutations were introduced in the endogenous copies of *Mll3* and *Mll4*, depleting global H3K4me1 levels [100, 107]. Whilst a reduction in cohesin binding at enhancers was observed [100], there were minimal effects on transcription, either of eRNAs or at target genes [107] and similar results were seen for MLL3/4 SET domain deletions [108]. Notably, only small reductions in H3K27ac at enhancers were observed with the point mutants, compared to the strong reductions in the double KO line [107]. Thus, the primary function of MLL3/4 at enhancers is likely independent of histone methylation, although how this is achieved is, as yet, unclear. It is worth noting, however, that whilst H3K4me1 levels were depleted they were not completely eliminated, leaving the importance of this histone modification in enhancer function still an open question.

### MLL family protein complexes

There is not sufficient space in this review to fully explore the detailed and diverse compositions of MLL family complexes. However, here we touch on some of the different complex components, with a particular focus on MLL, to illustrate how differences in complex composition may explain some of the phenotypic differences observed among family members.

The stable core complexes of MLL and MLL2 are highly similar with very little to differentiate them, although each protein displays additional protein interactions that may be suggestive of different functions of the two proteins. Both MLL and MLL2 are targeted for proteolytic cleavage by the threonine aspartase Taspase 1 [40, 137–139], generating 320-kDa N-terminal (MLL-N) and 180-kDa C-terminal (MLL-C) fragments, in the case of MLL (Fig. 2). These fragments do not dissociate after cleavage, however; the FYRN and FYRC domains interact to form a single domain, holding the complex together [140]. Interestingly, cleavage separates the C-terminal SET domain from the N-terminal portion, which contains all of the known binding domains of MLL.

As discussed above, the MLL SET domain is functional only when in complex with WDR5, RbBP5 and ASH2L [55]. In addition, MLL stably interacts with a number of other proteins to modulate its localisation and activity. The N-terminus of MLL is associated with two key factors, menin and LEDGF (lens epithelium-derived growth factor) [141–145]. Menin is required for the expression of MLL target genes, including *Hoxa9*, *Meis1, CDKN1B* (p27) and *CDKN2C* (p18) [72, 143, 146] and this interaction is necessary for MLL fusion protein-mediated leukemogenesis [143, 147]. The interaction with menin (although not LEDGF) is shared with the MLL2 protein [72], but not with any other members of the MLL family. The interaction between MLL and menin generates a binding pocket for LEDGF, thus generating a ternary complex [148]. LEDGF promotes transcriptional activation [149, 150] and is essential for leukemogenesis [144], suggesting that a key role of menin may be to bridge the interaction between the two proteins. This transcriptional coactivator function [149, 150] could be a unique aspect of MLL complex activity. At least in some experiments, menin and LEDGF do not appear to be required to recruit MLL to target genes, with the N-terminal, menin-interacting, region of MLL being dispensable for recruitment of MLL constructs to *Hoxa9* [151].

MLL has also been observed to interact with transcription cofactors, suggesting that a co-localisation of activities may be important for its function. For example, interactions have been identified between MLL and the lysine acetyltransferases (KATs) CBP [152], MOZ [153] and MOF [54], the latter of which is known to be important for MLL target gene expression, likely via H4K16 acetylation [54, 60]. Alone among CXXC domains, the domain within MLL has also been found to interact with the PAF1 complex [151, 154], which may provide a bridge to RNA pol II itself [155]. This again could be a unique aspect of MLL function, since MLL2 does not bind to the PAF1 complex [151].

Another distinct characteristic of the founder MLL is its negative regulation via interaction with repressive factors. Several proteins have been observed to immunoprecipitate with the CXXC domain region of MLL, originally defined as a repression domain due to the effects of these interactions on transcription [156]. Binding partners include the PcG proteins BMI-1 and HPC2, and the corepressors CtBP and HDAC1 [157]. In addition, the third PHD of MLL is bound by the cyclophilin CyP33 [158–161], and the extended PHD3/bromodomain region interacts with ASB, a substrate recognition subunit for the Elongin B/C-Cullin-SOCS box protein (ECS) E3 ubiquitin-ligase, targeting the methyltransferase for proteolysis during hematopoiesis [162].

MLL2 has very few unique known interacting partners, other than an interaction with AKAP95 [81]. The MLL3 and MLL4 proteins have multiple distinct interacting partners including the H3K27me3 demethylase UTX, PA1, PTIP and p53 [73, 163, 164]. However, there is very little that differentiates MLL3/4. Similarly, the SETD1A/B complexes are highly similar [47–49, 80], although the two proteins show distinct subnuclear distributions [49], indicating they are non-redundant. In addition to its apparently major role in promoting H3K4me3 at promoters in mammals, SETD1A also has an important non-SET role in regulating the DNA damage response [165]. Although unique complex components can help explain functional divergence of MLL family members, it is still difficult to fully explain why all six MLL family members are individually required for organism survival, producing distinct KO phenotypes.

## Recruitment of MLL family members

To some extent, the term recruitment can be misleading, as it would seem to imply a deterministic intent in directing proteins to where they need to go. Single-molecule tracking (SMT) experiments of transcription factor (TF) binding in *E. coli* show that free diffusion coupled with non-specific DNA-TF interactions directs TFs towards their high-affinity binding sites in a process termed facilitated diffusion [166]. Weak, non-specific interactions likely represent a general way in which DNA-binding factors are funnelled down an affinity gradient towards their high-affinity binding sites [167]. Chromatin proteins tend to lack high-affinity DNA-binding domains and are much more likely to contain a large number of low-affinity chromatin and DNA-binding domains [98, 168]. Interestingly, in mammalian SMT experiments, the behavior of chromatin-like proteins suggests that they never freely diffuse and are instead “trapped” by a large number of low-level weak interactions that cause them to slowly “creep” along the surface of chromatin [169]. This behavior is consistent with the notion of multivalency (discussed in [168]). Multivalent interactions appear to be relatively common for chromatin factors, where many of the binding domains have only a low affinity for their substrate. Combining the affinities of multiple interactions can not only increase the strength of binding, but can also create a higher stringency for localisation to sites at which multiple epitopes must be present. This is thought to be a major way in which chromatin proteins search chromatin and then create stable complexes at specific chromatin sites [98, 106, 151, 168]. This idea is also consistent with recent work on the involvement of chromatin proteins in the self-organizing formation of phase-separated condensates that generate distinct regulatory domains [170, 171].

A notable characteristic of the MLL family proteins is the presence of multiple chromatin-interaction domains (Fig. 2), suggesting that a multivalency effect may be responsible for their genomic association. All four MLL proteins contain multiple PHD fingers [56, 172], but they do not show the same interaction specificities. PHD3 of MLL preferentially binds H3K4me3 tails [151, 161, 173], allowing the KMT to recognize its own product, and this appears to be important for MLL localisation at individual target genes [151, 161, 173]. MLL also contains an atypical bromodomain adjacent to PHD3 that does not appear to interact with acetylated lysine residues, although it has low stability in vitro and a fully intact domain has been difficult to work with [161, 174].

MLL and MLL2 contain a CXXC domain [56, 175], a protein domain known to recognize non-methylated CG-rich DNA (CpG islands) [176–179]. Interestingly, although MLL and MLL2 overall share a highly similar domain architecture with *Drosophila* trx, trx itself does not include a CXXC domain [4, 10], likely because of the relative lack of CpG islands in *Drosophila* [180], suggesting some divergence in localisation mechanisms between the species. Recognition of unmethylated CpGs is thought to be an important localisation mechanism for MLL [151, 181], and is required for myeloid transformation by the MLL–ENL fusion protein [181]. Although SETD1A/B do not contain a CXXC domain, they reside in a complex with the CXXC domain-containing protein CFP1 [47–49] which stabilizes SETD1A/B activity at promoters [106]. MLL3 and MLL4, in contrast, do not possess CXXC domains, consistent with the fact that while most active gene promoters contain a CpG island [182], only a small proportion of enhancers are at CpG islands [183].

The MLL CXXC domain binds CpG DNA with a Kd of 4.3 µM [178], and the PHD3 affinity for H3K4me3 tails is similar (Kd 19 µM [173] or 4.3 µM [161]). Individually, these low µM interactions are unlikely to provide sufficient binding affinity to stabilize MLL binding at target loci, suggesting that the combination of the two interactions is necessary for an association with chromatin. In support of this, in an experiment involving recruitment of MLL-N fragments to the *HoxA9* locus in *Mll* KO MEFs, both the CXXC domain- and PHD finger-containing regions of the protein were required [151].

These domains cannot be sufficient for precise targeting of MLL, however; the same bivalent interaction (binding to unmethylated CpG and H3K4me3) is also used by the SETD1A/B complexes [106]. These are localized to active gene promoters via CFP1, which contains both a PHD and CXXC domain [48, 184, 185]. No single ChIP-seq dataset exists to compare the binding profiles of these different KMTs; however, by inference and in some cases by direct comparison, MLL, MLL2 and SETD1A/B all show partially distinct genomic distributions [85, 106, 120]. This strongly argues that despite the common binding motifs, additional activities are required to discriminate the binding profiles of these different proteins. For example, MLL and MLL2 contain additional DNA-binding motifs in the form of multiple HMG-like N-terminal AT hooks [156], which promote binding to AT-rich DNA.

The AT hook and menin/LEDGF interaction domains of MLL are dispensable for recruitment of MLL-N to *HoxA9* [151], although without a genome-wide analysis it is difficult to know whether this applies to all MLL-bound loci. LEDGF itself contains a PWWP domain with specificity for H3K36me2 [186–190]; whilst MLL and LEDGF binding are not observed at all sites of H3K36me2, this may provide an additional stabilizing interaction for the complex at chromatin. Indeed, MLL binding at several target genes is reduced upon knockdown of the H3K36 methyltransferase ASH1L, although this has only been specifically observed in MLL rearranged leukemias [190].

One additional possibility is that binding of MLL to other chromatin proteins, for example, the KATs p300/CBP, MOF and MOZ, may provide further stabilizing interactions at specific loci. Whilst it is possible to argue that MLL “recruits” these KATs to target genes, the reverse is also plausible; p300/CBP, for example, interact with a large number of transcription factors [191]. Indeed, MLL has been shown to bind to CBP cooperatively with CREB or MYB, suggesting a synergism with additional factors, which may enhance specificity [152, 192]. Further, given that the KATs themselves contain chromatin-interaction domains (for example, bromodomains [191]) a more nuanced model would be that together these proteins generate a network of relatively low-affinity interactions, stabilizing the complex as a whole at chromatin.

A more sequence-specific mechanism for MLL3/4 interaction with chromatin has been proposed. Ectopic expression of CEBPβ or HOXA9 is sufficient to generate novel enhancers, including binding of MLL3/4 and deposition of H3K4me1 [122, 193], suggesting the potential for transcription factor-mediated localisation. However, the mechanism behind this process has not yet been elucidated. In addition, MLL3/4 also contain multiple chromatin-binding domains, so additional stabilizing interactions may be involved. In contrast to the H3K4me3-binding MLL PHD3 domain, the tandem PHD4-6 region of MLL4 (see Fig. 2) recognizes the histone H4 N-terminal tail, dependent on the absence of symmetric dimethylation of H4R3 [194]. A further interaction between ePHD6, a fragment containing PHD6 and the preceding zinc finger, and histidine 18 of a histone H4 tail peptide, has also been demonstrated [195], indicating multiple contacts between MLL4 and histone H4. These interactions are required for the in vitro methyltransferase activity of MLL4, and mutation of PHD6 disrupts expression of MLL4-dependent genes in vivo [195], although it is not clear what effect, if any, this has on enhancer localisation. Overall, there is no clear and obvious mechanistic explanation for how each MLL family protein could be uniquely and specifically localized to gene targets without the inclusion of additional recruitment factors.

## Conclusions on the role of H3K4 KMT activity in MLL family activity

One of the questions we wanted to address is: why are there so many MLL KMTs in mammals? Since each protein is individually required for normal development, mammals have clearly evolved a need for all six MLL family members. However, the data are mixed on how important the individual KMT activities actually are. As discussed above, differential recruitment mechanisms of the MLL family do not seem to address this issue of specificity. Instead, differences in protein complex stoichiometry suggest that the requirement for each individual KMT could be determined to some extent by abundance [188]. This fits with the observation that SETD1A is the most abundant MLL family member [188] and it also appears to be responsible for most H3K4me3 in the cell [128]. If this forms part of the explanation, there must be some variation in stoichiometry across different tissues and/or stages of development; for example, MLL2 appears to be the major KMT responsible for H3K4me3 levels during oogenesis [196].

MLL KMT activity is not required for normal development or hematopoiesis [59, 60], but this contrasts with the observation that in *Drosophila*, point mutations in the trx SET domain are sufficient to cause *Hox* gene-mediated developmental defects [116]. Given the duplication of MLL family members in mammals, it is possible that some redundancy in H3K4 methyltransferase activity exists, despite their unique non-methyltransferase functions, so the effect of the MLL SET deletion is masked by the presence of MLL2 SET domain function [120]. This also argues that at least globally, the KMT activity is more crucial for some members than for others, and that many of the MLL proteins have evolved to have other functions. For example, an important role for MLL may be recruitment of MOF and the promotion of H4K16ac [54, 60]. Even SETD1A, which appears to be the major KMT in mammals, has additional activities not related to SET domain catalytic activity [165]. Alongside interactions with different protein complex members, it also remains possible that the MLL KMTs could have specificity for unique non-histone protein targets, such as methylation of p53 [197, 198].

One other key question is: how important is H3K4 methylation for the cell? In the case of MLL3/4, it seems clear that the proteins themselves are required for enhancer function, but MLL3/4-mediated H3K4me1 is not essential [107, 108]. The evidence is also fairly strong that H3K4me3 is not absolutely required for transcriptional activation or enhancer function [103], and in fact is not intrinsically required for cell survival [41]. The SET domain mutation in *Drosophila* trx suggests that although H3K4me3 may not be important for transcription or cell survival per se, it still could have a key role in normal development [116]. Similarly, some PcG mutations have modest effects on gene regulation in ES cells, but these same PcG mutant ES cells are unable to properly control gene expression transitions through differentiation [199]. This is consistent with the original observations of PcG and trxG mutations, where early development often proceeded normally, and only at later points did development and normal gene regulation begin to break down [15, 17]. It has been argued that the role of H3K4me3 is to reduce transcriptional noise and increase consistency between individual cells, rather than dictate the population mean gene expression level [103, 200]. As most analyses of H3K4me3 look at bulk population averages, these effects could have been missed in past experiments. Further, given the levels of cellular heterogeneity in a developing organism, this issue is likely of particular relevance compared to the steady state of cell lines.

One major function of histone marks, such as H3K4me3, then, may be to contribute to transcriptional memory, ensuring that active genes retain that state during successive cell divisions. Notably, Trx remains associated with DNA during S phase, suggesting that it may rapidly methylate nascent nucleosomes after deposition [201]. Interestingly, when unusual DNA structures disrupt DNA replication, histone marks are lost and gene regulation breaks down [202]. This fits with the potential role of H3K4 methylation in stabilizing promoter complex formation [79], especially through the cell cycle, thus increasing the probability that proper gene expression patterns are maintained. Indeed, histone marks are known to influence TF binding immediately following DNA replication [203]. In this way, H3K4me3 may provide less of an on–off switch than a way to decrease the stochastic nature of complex formation, thus ensuring the stability of gene expression patterns. This may explain why these marks and proteins are so often mutated in cancer, where increased stochasticity may make advantageous changes more likely in the expression of genes regulating proliferation and differentiation. Importantly, such a model would also explain why these systems are only absolutely crucial during the highly dynamic processes of multi-cellular organism development.
